# Follow-up analysis of voice quality in patients with late-onset Pompe disease

**DOI:** 10.1186/s13023-018-0932-1

**Published:** 2018-10-26

**Authors:** Krzysztof Szklanny, Anna Tylki-Szymańska

**Affiliations:** 1 Multimedia Department, Polish-Japanese Academy of Information Technology, Warsaw, Poland; 2 Department of Paediatrics, Nutrition and Metabolic Diseases, The Children’s Memorial Health Institute in Warsaw, Warsaw, Poland

**Keywords:** Pompe disease, Metabolic myopathy disorders, Voice quality, Electroglottography, Acoustic methods, Vocal folds, Voice disorders

## Abstract

**Background:**

Late-onset Pompe disease (LOPD) is a metabolic myopathy disorder characterized by progressive muscle damage and among others dysfunction of the voice apparatus, which affects speech and – above all – voice quality. Symptoms include dysphonia, instability, glottic insufficiency, and tense voice. The aim of this study was to evaluate and compare voice quality disorder in a group of 15 LOPD patients who were first examined in 2014 and then re-examined in 2017.

**Methods:**

In both 2014 and 2017, the same 15 LOPD patients, ranging in age from 15 to 57, from 10 different families, underwent the following examinations: perceptual assessment of voice quality on the RBH scale, electroglottographic recordings, and acoustic recordings. All the patients were on enzyme replacement therapy (ERT).

**Results:**

Three years after the 2014 study, the LOPD patients demonstrated a deterioration in voice quality. A statistically significant increase in glottic insufficiency (*p* = 0.0399) and a shift towards tense voice (*p* = 0.0417) were observed. Two patients – out of three who had received presymptomatic treatment – demonstrated stable voice quality compared with 2014.

**Conclusions:**

The results suggest increased muscle weakness and progression of LOPD. The parameters Closed Quotient (calculated on the basis of an electroglottographic signal) and Peak Slope (calculated on the basis of an acoustic signal) proved to be the most sensitive.

## Background

Pompe disease (glycogen storage disease type II, GSD II) is a progressive metabolic myopathy caused by a deficiency of lysosomal alpha-glucosidase. This leads to an accumulation of glycogen, mainly in the muscles, causing their progressive impairment. The spectrum of clinical phenotypes includes an infantile form (classic form) and a late-onset form (with both juvenile and adult presentation). In the juvenile form, the first symptoms – such as progressive proximal and axial muscle weakness – appear between 2 to 5 years of age [1–3]. The late-onset is characterized by a slow progression. The disease has a particularly damaging effect on the functioning of the skeletal muscles, with sufferers eventually confined to a wheelchair and requiring ventilator-assisted breathing. Disease progression presents increasingly clearer clinical manifestations of cell damage, with dysfunction developing in the voice apparatus.

This dysfunction presents both speech and voice impairment and includes: articulation problems, dysarthria, consonant substitutions, consonant omissions and cluster reductions, mild to moderate hypernasal resonance, velopharyngeal incompetence, hoarseness, dysphonia, glottic insufficiency, and tense voice [3–11].

The aim of this study was to evaluate voice quality disorder in a group of patients with late-onset Pompe disease (LOPD), comparing test results obtained in 2014 with those from 2017. Both examinations involved the same patients and the same voice parameters. Voice quality assessment was carried out using acoustic and electroglottographic analysis.

## Methods

The 2017 examination was performed on 15 patients, with an age range of 15–57 (mean age of 30.8). The patients came from 10 different families. The same 15 patients had previously been examined in a similar study in 2014. All the patients were on enzyme replacement therapy (ERT). The patients’ clinical data, mutations, and the length of ERT are shown in Table 1.

**Table 1:** Patient demographics

ID	Gender	Current age years	Age of first symptoms years	Mutation	Years on ERT
1	F	14.8	no symptoms, family screening	IVS1-13 T > G/c.2662G > T	10
2	M	18.8	no symptoms, family screening	IVS1-13 T > G/c.2662G > T	10
3	M	28.6	6	IVS1-13 T > G/c.2662G > T	10
4	F	10.5	1	2495delCA(ex18)/2495 delCA (ex18)	9.5
5	M	11.5	no symptoms, family screening	G377S c.2495_2496 delCA	9
6	F	17.8	3	C1129G > A/c.2495_2496 delCA	11
7	M	20.8	3.5	IVS1-13 T > G/c.925G > A	10
8	F	43	6	c.364A > G/c.1796C > T	10
9	F	34	7	IVS1-13 T>/C103G	10
10	M	40.5	27	c.364A > G/c.1796C > T	10
11	M	49.5	35	IVS1-13 T > G/c.307 T > G	10
12	M	37.8	15	IVS1-13 T > G/c.307 T > G	10
13	M	56.8	33	IVS1-13 T > G/C103G, c.307 T > G	11
14	M	40.8	28	IVS1-13 T > G/c.307 T > G	8
15	F	36.8	26	IVS1-13 T > G/c.307 T > G	8

Set 1 refers to the results for the 15 patients when first examined in 2014, whereas Set 2 refers to the results for the same 15 patients when re-examined in 2017. All 15 patients participated in identical acoustic and electroglottographic recordings in both 2014 and 2017.

The examinations were supplemented by voice quality assessments based on a perceptual assessment of voice quality on the RBH scale [12, 13]. The RBH perceptual scale is used in German clinics and is recommended by the Committee on Phoniatrics of the European Laryngological Society.

- R - Rauigkeit (roughness) – the degree of voice roughness resulting from irregular vocal fold vibrations
- B - Behauchtheit (breathiness) – the degree of breathiness resulting from glottic insufficiency
- H - Heiserkeit (grade of hoarseness)

The RBH scale assigns scores of 0, 1, 2, and 3 for all parameters, depending on the degree of vocal disorder, where ‘0’ means normal voice, ‘1’ – a slight degree, ‘2’ – a medium degree, and ‘3’ – a high degree. Perceptual assessment of patient voice quality was carried out on both occasions by the same two independent experts. The evaluators were blinded. Both experts possess many years of professional experience in voice/speech signal assessment and have completed the RBH learning course. Dejonckere et al. [14] confirmed that the GRB parameters and the German RBH scale (R = Rauhigkeit = Roughness, B = Behauchtheit = Breathiness, and H = Heiserkeit = Hoarseness) have equivalent clinical value.

The study was approved by the Bioethics Committee (133/KBE/2014) of the Children’s Memorial Health Institute in Warsaw. All participants gave informed, written consent prior to their participation, and this consent was approved by the committee. Consent on behalf of the children enrolled was in writing.

## Methods for voice quality analysis

The study was carried out with an EG2-PCX2 electroglottograph, a Glottal Enterprises measurements device. For the electroglottographic recordings, the patients phonated with a neutral fundamental frequency F_0_, at a sound pressure level of 55–80 dBA.

### Electroglottography

Electroglottography (EGG) is a noninvasive technique used to register vocal fold vibrations by measuring varying impedance across the throat during phonation [15–17]. An EGG recording enables a thorough phoniatric examination, allowing for the detection of abnormalities in the patient’s voice [18, 19].

Measurements were taken using a Glottal Enterprises EG2-PCX2 electroglottograph device. Two electrodes were placed on either side of the subject’s neck at the level of the larynx. A constant amplitude high-frequency voltage (2 MHz) with a maximum interelectrode voltage of 1.5 V was applied. This setup was chosen to ensure conditions standard for clinical applications. The acoustic analysis was performed with an attached ECM 8000 Behringer microphone with a 48 kHz sampling frequency and 16-bit depth, and then resampled to 16 kHz; this is adequate for speech/voice analysis. For the purposes of EGG signal analysis, the CQ H (Closed Quotient) parameter, proposed by Howard [20, 21] was calculated. CQ H shows the percentage of each cycle when the vocal folds are in contact. It is a hybrid calculation, using the EGG contacting peak for detecting the glottal contact event, and an EGG-based 3/7 threshold for detecting the glottal opening event.

### Acoustic analysis parameters

For the purposes of the acoustic analysis, the microphone signal obtained in the electroglottographic recordings was used. The vowel /a:/ was selected for analysis, as it is easy to pronounce and has distinctive formant features (spectral energy maxima) distinguishing it from other phonemes [22]. The vowel was phonated three times, for a sustained period, at a sound pressure level of 55–80 dBA. Each patients’ vowel phonation /a:/ lasted from 6 to 12 s. Of the three phonations, those that failed to sound - e.g. due to accidental coughing - were rejected. Phonations with the least audible changes in voice were selected for further processing.

These recordings were used to assess vocal fold vibration and voice quality. The MATLAB (COVAREP toolkit) [23] was used for further analysis of Peak Slope, Normalized Amplitude Quotient, Cepstral Peak Prominence, and Harmonic Richness Factor parameters.

**Peak Slope (PS)** makes it possible to effectively monitor changes in voice quality and to distinguish between breathy, modal, and tense voice [23–25]. The main advantage of the Peak Slope algorithm is that it functions as a standalone program independent of other algorithms.

**Normalized Amplitude Quotient (NAQ)** is presented as a method to parametrize the glottal closing phase using two amplitude-domain measurements from waveforms. NAQ has been used to effectively separate types of phonation [26].

**Cepstral Peak Prominence (CPP**) parameter allows for the detection of early dysphonia. CPP is a robust voice analysis algorithm that measures the degree of harmonic structure within a voice signal. It has been shown to correlate well with perceptions of breathiness, because it is a measure of periodicity [27, 28]. A normal voice, which has a well-defined harmonic structure, will have a strong cepstral peak. In breathy voice, the entire cepstrum is relatively flat, resulting in a low CPP value.

**Harmonic Richness Factor** (**HRF)** parameter permits the detection of dysphonia, as it helps identify the structure of harmonic components in speech signals. This parameter is calculated as a ratio of consecutive harmonics, from second to first [29].

## Results

The perceptual assessment of voice quality on the RBH scale is shown in Table 2. The acoustic and electroglottographic analysis results for LOPD patients are shown in Table 3.

**Table 2:** RBH scale results

	RBH Set 1	RBH Set 2	
	101	212	
	101	101	
	101	101	
	000	000	
	000	001	
	020	020	
	111	110	
	000	000	
	100	000	
	000	000	
	101	101	
	000	000	
	000	000	
	000	000	
	000	000	

R = degree of voice roughness caused by irregular vibrations of the vocal folds; B = degree of breathy voice caused by air due to glottic insufficiency; H = degree of hoarseness. Each parameter has a value in the range 0–3. A smaller value indicates better quality of voice. “0” stands for normal voice

**Table 3:** Mean values and standard deviation of CQ H, PS, NAQ, CPP, HRF values for all Sets

Parameter/Set	Set 1	Set 2	*p* value
CQ H	0.345 ± 0.070	0.307 ± 0.047	0.0399*
PS	− 0.304 ± 0.153	−0.372 ± 0.109	0.0417*
NAQ	0.122 ± 0.033	0.138 ± 0.040	0.2062
CPP	11.476 ± 0.467	11.403 ± 0.529	0.6487
HRF	22.057 ± 8.718	23.254 ± 8.504	0.1579

The results are reported as Mean ± Standard Deviation; t-Test results are also reported

* Statistically significant

*CQ H* Closed Quotient, *PS* Peak Slope, *NAQ* Normalized Amplitude Quotient, *CPP* Cepstral Peak Prominence, *HRF* Harmonic Richness Factor

### Statistical analysis results

Comparison of the accuracy and reliability of perceptual voice quality scale annotation on the RBH scale was performed by the two experts using non-parametric tests as the distribution for the RBH scale is not normal. For parameters R, B, and H, the differences in annotation were statistically insignificant.

Anderson-Darling and Saphiro-Wilk tests were used to calculate normal distribution for each parameter. All the acoustic parameters were distributed normally. Differences in the values of parameters PS, NAQ, CPP, HRF, and CQ H were calculated using independent samples *t*-tests. F-tests were used to check whether variances were equal for each parameter. Statistical analysis was carried out in the MATLAB environment.

### The RBH scale results

Voice quality disorder was observed in seven patients in Set 1 and seven patients in Set 2 (Table 2). Two patients from Set 2 demonstrated an improvement in voice quality (patients 7 and 9), and another two patients from Set 2 demonstrated deterioration in voice quality (patients 1 and 5).

Rough voice was observed in six patients from Set 1 (patients 1, 2, 3, 7, 9, 11) and five patients from Set 2 (patients 1, 2, 3, 7, 11). Breathy voice was observed in two patients from Set 1 (patients 6, 7) and three patients from Set 2 (patients 1, 6, 7). Hoarse voice was observed in five patients from Set 1 (patients 1, 2, 3, 7, 11) and five patients from Set 2 (patients 1, 2, 3, 5, 11). Changes in the assessment of voice quality were noticeable in individual patients, but the differences in Paired Wilcoxon Signed Rank results between Sets 1 and 2 were statistically insignificant (R – test statistic = 1.5, *p* = 1; B - test statistic = 0 p = 1; H - test statistic = 2, p = 1).

### Electroglottographic analysis results (Fig. 1)

Glottic insufficiency was observed in 13 patients from Set 1 and in all 15 patients in Set 2. Deterioration was observed in patients 6 and 9 from Set 2. The mean value of the CQ H parameter in Set 1 was higher than that in Set 2 (0.345 vs 0.307), indicating increased glottic insufficiency since 2014. The difference is statistically significant (*t* = 2.265, p =  0.025).

**Fig. 1:**
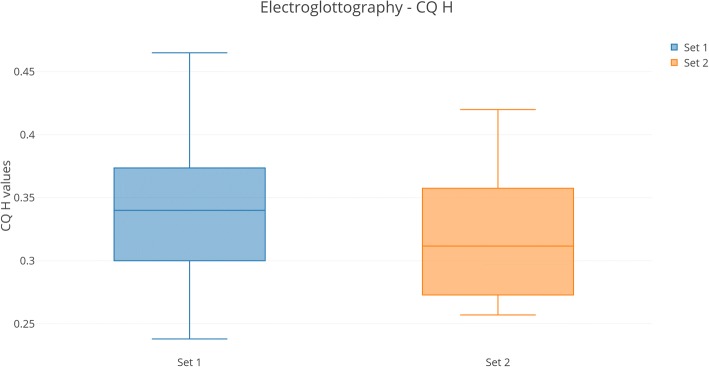
Visualization of electroglottographic values for CQ H. Left: graph for LOPD patients in 2014. Right: graph for LOPD patients in 2017

### Acoustic analysis results (Fig. 2)

In Set 1, the Peak Slope parameter indicated the presence of tense voice in seven patients (patients 2, 3, 7, 8, 11, 12, 13), whereas in Set 2 tense voice was found in 11 patients (patients 2, 3, 6, 7, 8, 9, 11, 12, 13, 14, 15). Therefore, the mean Peak Slope value was lower (− 0.304 vs − 0.372). The difference is statistically significant.

**Fig. 2:**
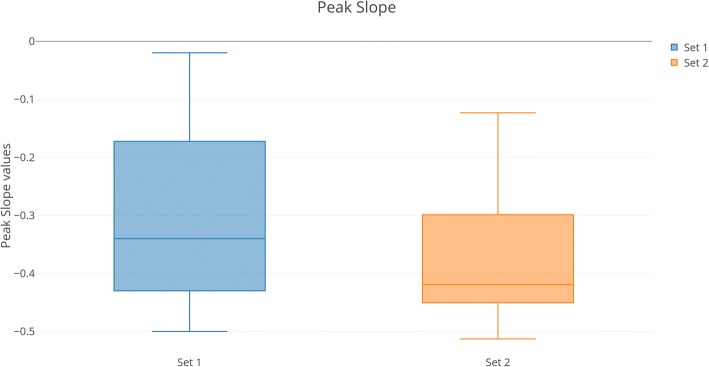
Visualization of acoustic data for Peak Slope. Left: graph for LOPD patients in 2014. Right: graph for LOPD patients in 2017

NAQ (*t* = 1.326, *p* =  0.2062), CPP (t = 0.465, *p* =  0.6487) and HRF (t = 1.492, *p* =  0.1579) showed differences in parameter values. These differences indicate deterioration in voice quality, although they are not statistically significant.

## Discussion

In recent years, many studies have examined patients with the late-onset type of Pompe disease for speech quality disorder [3, 5, 7, 9]. However, to-date, only one study has assessed voice quality deviations in patients with LOPD [9].

Our 2014 study indicated voice quality disorders. The symptoms observed in Set 1 included dysphonia, glottic insufficiency, tense voice, voice pitch fluctuation, and variation within the same phonation. Acoustic analysis and EGG results indicated deviations in voice quality more clearly than by video laryngoscopic examination. Voice quality disorder included glottic insufficiency and tense voice [9]. The results of the assessments carried out three years later showed further deviations in the functioning of the vocal apparatus, including the progression of glottic insufficiency. This was found in 13 patients from Set 1 and in all 15 patients from Set 2. The difference is statistically significant (Table 3). Clinically, the condition of patients did not change significantly between 2014 and 2017.

Nine patients demonstrated increased glottic insufficiency. This could indicate increased muscle weakness as the disease progressed. The RBH scale also indicated voice deterioration over the intervening three year period. Breathy voice was observed in two patients from Set 1 (patients 6, 7) and three patients from Set 2 (patients 1, 6, 7).

Peak Slope indicated tense voice in seven patients from Set 1 and patients from Set 2. This difference is also statistically significant. Nine patients with previously diagnosed tense voice demonstrated considerable deterioration in this respect (Table 3). Perceptual assessment using the RBH scale proved less accurate, indicating deterioration of voice quality in only two patients.

Patients 2 and 5 began treatment in childhood during the presymptomatic phase. In these patients, in 2017 we observed stable voice quality compared with 2014. Perceptual evaluation indicated stable voice quality in one patient (2) compared with 2014. Although these patients voices were still characterized by abnormalities typical of Pompe disease. This conclusion matches the results obtained by other authors [30, 31]. The results presented in such studies indicate that the application of early diagnostics, such as screening tests for new-borns – as well as early treatment – can benefit infants with infantile-onset Pompe disease.

An improvement in voice quality – compared with 2014 – was observed in two patients using the RBH scale. However, acoustic analysis confirmed this in only one patient – 7. This patient had been diagnosed by chance at the age of 6 years. Previous biochemical tests on him had found elevated levels of liver transaminases and creatinine kinase, which recommended testing for Pompe disease. Enzymatic examination confirmed suspicions. The patient was therefore diagnosed with Pompe disease faster – before the onset of symptoms and subsequent treatment. The improvement in voice quality may also be related to his voice breaking, as he entered puberty in 2014. By 2017, his voice had fully broken, which is why hoarse voice decreased. In the second case, patient number 9, acoustic analysis did not confirm voice quality improvement.

A limitation of this study was the small group of patients with LOPD. In further studies, the size of groups should be increased. However, one advantage was that the same patients were examined. Also, there was no control group at work, as we compared the results with the previous study and verified against the parameter norms [24, 26]. When considering electroglottographic and acoustic analyses, attention should be paid to the signal analysis process, rejecting accidental incorrect phonations. Notwithstanding this, both forms of analysis remain objective methods for voice quality assessment. Perceptual voice evaluation should be supported by objective, automatic methods.

EGG and acoustic analysis found that not all parameters were equally sensitive. CQ H and PS proved to be the most sensitive to changes. It follows that selecting parameters to assess voice quality should depend on the disease and vary depending on the particular symptoms in the vocal tract [9, 24, 25]. NAQ, CPP, and HRF proved to be less sensitive and did not show statistical differences in relation to the previous study. The results and sensitivity of analyses performed in this study allowed objective demonstration of damage to vocal apparatus muscles.

## Conclusions

Changes in voice quality in LOPD patients were demonstrated, with deterioration in voice quality observed three years after the 2014 study. The changes in patients included increases in both tense voice and glottic insufficiency.

The poorer results obtained by EEG analysis may indicate increased muscle weakness and Pompe disease progression while on enzyme replacement therapy. The Closed Quotient H and Peak Slope parameters proved to be the most sensitive to change. Two patients – out of three who began ERT during the presymptomatic phase – demonstrated stable voice quality compared to 2014.

## Abbreviations

COVAREP: A Cooperative Voice Analysis Repository for Speech Technologies
CPP: Cepstral Peak Prominence
CQ H: Closed Quotient
EGG: Electroglottography
ERT: Enzyme replacement therapy
HRF: Harmonic richness factor
LOPD: late-onset Pompe disease
NAQ: Normalized Amplitude Quotient
PS: Peak Slope
